# (2-Hydroxy­ethyl)(prop­yl)aza­nium 2-[(2-carboxy­phen­yl)disulfan­yl]benzoate monohydrate

**DOI:** 10.1107/S1600536810007658

**Published:** 2010-03-06

**Authors:** Grant A. Broker, Edward R. T. Tiekink

**Affiliations:** a 5959 FM 1960 Road West, Houston, Texas 77069, USA; b Department of Chemistry, University of Malaya, 50603 Kuala Lumpur, Malaysia

## Abstract

With the exception of the terminal hydr­oxy group [N—C—C—O = 53.8 (5)°], the cation of the title salt hydrate, C_5_H_14_NO^+^·C_14_H_9_O_4_S_2_^−.^H_2_O, is a straight chain. A twisted conformation is found for the anion [C—S—S—C = −87.44 (16)°]. In the crystal, the anions self-assemble into a helical supra­molecular chain *via* charge-assisted O—H⋯O_c_ hydrogen bonds. These chains are connected into a three-dimensional network *via* N—H⋯O_c_, N—H⋯O_w_, O_h_—H⋯O_cb_, and O_w_—H⋯O_c_ hydrogen-bonding inter­actions (c = carboxyl­ate, w = water, h = hydr­oxy and cb = carbon­yl).

## Related literature

For related studies on co-crystal/salt formation involving 2-[(2-carboxy­phen­yl)disulfan­yl]benzoic acid, see: Broker & Tiekink (2007 ▶); Broker *et al.* (2008 ▶). For software for searching the Cambridge Structural Database, see: Bruno *et al.* (2002 ▶).
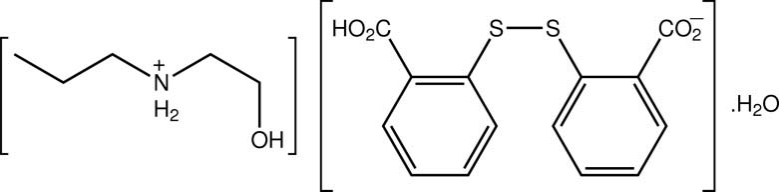


## Experimental

### 

#### Crystal data

C_5_H_14_NO^+^·C_14_H_9_O_4_S_2_^−^·H_2_O
*M* *_r_* = 427.52
Monoclinic, 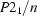

*a* = 8.1207 (16) Å
*b* = 17.714 (4) Å
*c* = 14.483 (3) Å
β = 99.58 (3)°
*V* = 2054.3 (7) Å^3^
*Z* = 4
Mo *K*α radiation
μ = 0.30 mm^−1^
*T* = 173 K
0.20 × 0.20 × 0.05 mm

#### Data collection

Rigaku AFC12/SATURN724 diffractometer
Absorption correction: multi-scan (*ABSCOR*; Higashi, 1995 ▶) *T*_min_ = 0.803, *T*_max_ = 1.000
12346 measured reflections
3600 independent reflections
3316 reflections with *I* > 2σ(*I*)
*R*_int_ = 0.051

#### Refinement

*R*[*F* ^2^ > 2σ(*F* ^2^)] = 0.068
*wR*(*F* ^2^) = 0.155
*S* = 1.17
3600 reflections
265 parameters
5 restraints
H-atom parameters constrained
Δρ_max_ = 1.11 e Å^−3^
Δρ_min_ = −0.43 e Å^−3^

### 

Data collection: *CrystalClear* (Rigaku/MSC, 2005 ▶); cell refinement: *CrystalClear*; data reduction: *CrystalClear*; program(s) used to solve structure: *SHELXS97* (Sheldrick, 2008 ▶); program(s) used to refine structure: *SHELXL97* (Sheldrick, 2008 ▶); molecular graphics: *ORTEPII* (Johnson, 1976 ▶) and *DIAMOND* (Brandenburg, 2006 ▶); software used to prepare material for publication: *publCIF* (Westrip, 2010 ▶).

## Supplementary Material

Crystal structure: contains datablocks global, I. DOI: 10.1107/S1600536810007658/pb2024sup1.cif

Structure factors: contains datablocks I. DOI: 10.1107/S1600536810007658/pb2024Isup2.hkl

Additional supplementary materials:  crystallographic information; 3D view; checkCIF report

## Figures and Tables

**Table 1:** Hydrogen-bond geometry (Å, °)

*D*—H⋯*A*	*D*—H	H⋯*A*	*D*⋯*A*	*D*—H⋯*A*
O2—H2*o*⋯O3^i^	0.84	1.70	2.535 (4)	178
N1—H1n⋯O4^ii^	0.90	2.10	2.887 (4)	146
N1—H2n⋯O1w^iii^	0.90	1.92	2.773 (5)	158
O5—H5o⋯O1^iv^	0.84	1.94	2.751 (5)	162
O1w—H1w⋯O4^v^	0.84	1.99	2.823 (5)	174
O1w—H2w⋯O3	0.84	2.26	3.036 (5)	154

Symmetry codes: (i) 
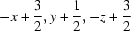
; (ii) 
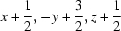
; (iii) 
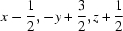
; (iv) 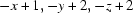; (v) 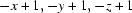.

**Fig. 1.**
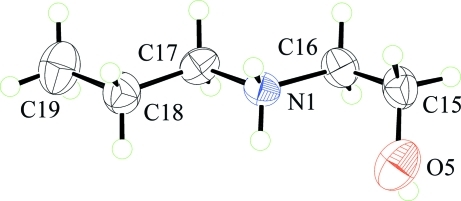
Molecular structure of the cation in (I) showing atom-labelling scheme and displacement ellipsoids at the 70% probability level.

**Fig. 2.**
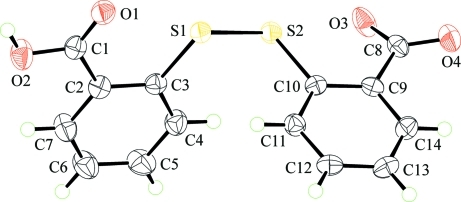
Molecular structure of the anion in (I) showing atom-labelling scheme and displacement ellipsoids at the 70% probability level.

**Fig. 3.**
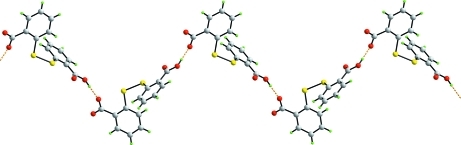
Supramolecular chain formation in (I) mediated by charge-assisted O–H···O- (orange dashed lines) hydrogen bonding. Colour code: S, yellow; O, red; N, blue; C, grey; H, green.

## supplementary crystallographic information

### Comment

The title salt hydrate, (I), was obtained during crystallisation experiments involving various N-containing species with the dicarboxylic acid, 2-[(2-carboxyphenyl)disulfanyl]benzoic acid (Broker & Tiekink, 2007; Broker *et al.*, 2008). The asymmetric unit of (I) comprises an aminium cation, Fig. 1, a uninegative 2,2'-dithio(benzoic acid)benzoate anion, Fig. 2, and a solvent water molecule of crystallisation.

The cation is linear with the exception of the terminal hydroxyl group which is twisted out of the chain as seen in the O5–C15–C16–N1 torsion angle of 53.8 (5) °. Confirmation of protonation of the amine-N1 atom during crystallisation is seen in the pattern of hydrogen bonding interactions, see below. A search of the CSD (Bruno *et al.*, 2002) suggests that this is the first structural characterisation reported for the 2-hydroxyethyl(propyl)aminium cation. The 2,2'-dithio(benzoic acid)benzoate molecule is twisted [C3–S1–S2–C10 = -87.44 (16) °] in accord with expectation with the conformation stabilised by intramolecular S···O interactions of 2.633 (3) Å for S2···O3 and 2.647 (3) Å for S3···O4 (Broker & Tiekink (2007). Confirmation that the C8—O1, O2 residue is in the acid form is found in the disparity of the C1–O1 and C1–O2 bond distances, *i.e*. 1.212 (4) and 1.316 (4) Å, respectively, compared with the equivalence of the C8–O3 and C8–O4 bond distances of 1.266 (4) and 1.247 (4) Å, respectively. The O1-carboxylic acid group is co-planar with the benzene ring to which it is connected with the C3–C2–C1–O1 and torsion angle being -2.9 (5) Å, but the O3-carboxylate group is not: the O3–C8–C9–C10 torsion angle being -21.0 (5) °.

The most distinctive feature of the crystal packing is supramolecular chain formed via self-association between 2,2'-dithio(benzoic acid)benzoate anions, as normally seen for such species (Broker & Tiekink, 2007), Fig. 2 and Table 1. The chain has a helical topology being generated by 2_1_-screw symmetry along the *b* axis of the monoclinic unit cell. The next most prominent O–H···O interaction involves the O5-hydroxyl group and the carbonyl-O1 atom. The remaining carboxylate-O4 associates with the water molecule of crystallization. The water molecule also forms a hydrogen bond with a neighbouring carboxylate-O3 atom and with a centrosymmetrically related water molecule to form a 12-membered {···OCO···HOH}_2_ synthon. The aminium-H2n atom forms a donor interaction to a O1w-water molecule so that the latter participates in three hydrogen bonds. The second ammonium-H1n atom forms a N—H···O hydrogen bond with the carboxylate-O4 atom which is also connected to the O1w-water molecule and therefore closes a 12-membered centrosymmetric {···O···HNH···OH}_2_ synthon. In this way the components of the crystal structure are linked into a 3-D network, Table 1.

### Experimental

The title salt (I) was obtained by dissolving 2-[(2-carboxyphenyl)disulfanyl]benzoic acid (0.100 g, Fluka) in ethanol (20 ml) to which was added the amine in 1:1, 1:2 and 1:3 stoichiometric ratios in three separate experiments. Regardless of the stoichiometry, only crystals of (I) were harvested as proved by multiple unit cell determinations, m.pt. 429–431 K

### Refinement

The H-atoms located from difference maps but placed in their idealised positions (O–H = 0.84 Å, N–H = 0.90 Å, and C–H 0.93-0.97 Å) and were included in the refinement in the riding model approximation with *U*_iso_(H) set to 1.2-1.5*U*_eq_(carrier atom). The maximum and minimum residual electron density peaks of 1.11 and 0.43 e Å^-3^, respectively, were located 0.91 Å and 0.60 Å from the H15b and O1w atoms, respectively.

### Crystal data

**Table:**

C_5_H_14_NO^+^·C_14_H_9_O_4_S_2_^−^·H_2_O	*F*(000) = 904
*M**_r_* = 427.52	*D*_x_ = 1.382 Mg m^−^^3^
Monoclinic, *P*2_1_/*n*	Mo *K*α radiation, λ = 0.71073 Å
Hall symbol: -P 2yn	Cell parameters from 6664 reflections
*a* = 8.1207 (16) Å	θ = 3.4–30.5°
*b* = 17.714 (4) Å	µ = 0.30 mm^−^^1^
*c* = 14.483 (3) Å	*T* = 173 K
β = 99.58 (3)°	Prism, colourless
*V* = 2054.3 (7) Å^3^	0.20 × 0.20 × 0.05 mm
*Z* = 4	

### Data collection

**Table:**

Rigaku AFC12K/SATURN724 diffractometer	3600 independent reflections
Radiation source: fine-focus sealed tube	3316 reflections with *I* > 2σ(*I*)
graphite	*R*_int_ = 0.051
ω scans	θ_max_ = 25.0°, θ_min_ = 3.4°
Absorption correction: multi-scan (*ABSCOR*; Higashi, 1995)	*h* = −9→9
*T*_min_ = 0.803, *T*_max_ = 1.000	*k* = −21→19
12346 measured reflections	*l* = −17→16

### Refinement

**Table:**

Refinement on *F*^2^	Primary atom site location: structure-invariant direct methods
Least-squares matrix: full	Secondary atom site location: difference Fourier map
*R*[*F*^2^ > 2σ(*F*^2^)] = 0.068	Hydrogen site location: inferred from neighbouring sites
*wR*(*F*^2^) = 0.155	H-atom parameters constrained
*S* = 1.17	*w* = 1/[σ^2^(*F*_o_^2^) + (0.0515*P*)^2^ + 2.9819*P*] where *P* = (*F*_o_^2^ + 2*F*_c_^2^)/3
3600 reflections	(Δ/σ)_max_ = 0.001
265 parameters	Δρ_max_ = 1.11 e Å^−^^3^
5 restraints	Δρ_min_ = −0.43 e Å^−^^3^

### Special details

**Table:**

Geometry. All esds (except the esd in the dihedral angle between two l.s. planes) are estimated using the full covariance matrix. The cell esds are taken into account individually in the estimation of esds in distances, angles and torsion angles; correlations between esds in cell parameters are only used when they are defined by crystal symmetry. An approximate (isotropic) treatment of cell esds is used for estimating esds involving l.s. planes.
Refinement. Refinement of *F*^2^ against ALL reflections. The weighted *R*-factor wR and goodness of fit *S* are based on *F*^2^, conventional *R*-factors *R* are based on *F*, with *F* set to zero for negative *F*^2^. The threshold expression of *F*^2^ > σ(*F*^2^) is used only for calculating *R*-factors(gt) etc. and is not relevant to the choice of reflections for refinement. *R*-factors based on *F*^2^ are statistically about twice as large as those based on *F*, and *R*- factors based on ALL data will be even larger.

### Fractional atomic coordinates and isotropic or equivalent isotropic displacement parameters (Å^2^)

**Table:**

	*x*	*y*	*z*	*U*_iso_*/*U*_eq_	
S1	0.69957 (11)	0.89493 (5)	0.68210 (6)	0.0297 (3)	
S2	0.63482 (11)	0.78938 (5)	0.62977 (6)	0.0301 (3)	
O1	0.8096 (3)	1.01510 (14)	0.78455 (18)	0.0366 (6)	
O2	1.0446 (3)	1.07927 (16)	0.7855 (2)	0.0442 (7)	
H2o	1.0216	1.1032	0.8317	0.066*	
O3	0.5300 (3)	0.65217 (14)	0.5766 (2)	0.0436 (7)	
O4	0.2893 (3)	0.62652 (15)	0.48483 (19)	0.0426 (7)	
O5	0.4552 (5)	0.9986 (2)	1.1203 (3)	0.0670 (10)	
H5O	0.3620	0.9926	1.1373	0.101*	
O1W	0.6571 (5)	0.5265 (2)	0.4645 (3)	0.0763 (11)	
H1W	0.6755	0.4821	0.4838	0.114*	
H2W	0.5987	0.5504	0.4974	0.114*	
N1	0.4341 (4)	0.87963 (18)	0.9850 (2)	0.0387 (8)	
H1N	0.5317	0.8935	0.9685	0.046*	
H2N	0.3630	0.9187	0.9728	0.046*	
C1	0.9391 (4)	1.0248 (2)	0.7550 (2)	0.0300 (8)	
C2	0.9888 (4)	0.9776 (2)	0.6796 (2)	0.0298 (8)	
C3	0.8909 (4)	0.9169 (2)	0.6417 (2)	0.0274 (7)	
C4	0.9445 (5)	0.8745 (2)	0.5709 (3)	0.0353 (9)	
H4	0.8798	0.8342	0.5443	0.042*	
C5	1.0921 (5)	0.8913 (2)	0.5397 (3)	0.0428 (10)	
H5	1.1263	0.8624	0.4928	0.051*	
C6	1.1889 (5)	0.9512 (2)	0.5783 (3)	0.0444 (10)	
H6	1.2884	0.9627	0.5575	0.053*	
C7	1.1376 (5)	0.9937 (2)	0.6473 (3)	0.0386 (9)	
H7	1.2030	1.0339	0.6731	0.046*	
C8	0.4090 (4)	0.66936 (19)	0.5131 (2)	0.0286 (8)	
C9	0.4176 (4)	0.74599 (19)	0.4703 (2)	0.0264 (7)	
C10	0.5159 (4)	0.80430 (18)	0.5153 (2)	0.0238 (7)	
C11	0.5186 (4)	0.8742 (2)	0.4707 (2)	0.0292 (8)	
H11	0.5838	0.9132	0.5001	0.035*	
C12	0.4245 (4)	0.8856 (2)	0.3827 (2)	0.0324 (8)	
H12	0.4263	0.9325	0.3540	0.039*	
C13	0.3281 (5)	0.8281 (2)	0.3371 (2)	0.0352 (9)	
H13	0.2664	0.8357	0.2777	0.042*	
C14	0.3250 (4)	0.7591 (2)	0.3812 (2)	0.0330 (8)	
H14	0.2598	0.7204	0.3510	0.040*	
C15	0.5417 (6)	0.9308 (3)	1.1412 (3)	0.0498 (11)	
H15A	0.5489	0.9203	1.2075	0.060*	
H15B	0.6546	0.9367	1.1286	0.060*	
C16	0.4617 (6)	0.8648 (2)	1.0865 (3)	0.0474 (11)	
H16A	0.5326	0.8207	1.0999	0.057*	
H16B	0.3555	0.8538	1.1059	0.057*	
C17	0.3663 (5)	0.8148 (2)	0.9263 (3)	0.0427 (10)	
H17A	0.2652	0.7974	0.9469	0.051*	
H17B	0.4467	0.7739	0.9356	0.051*	
C18	0.3278 (6)	0.8329 (3)	0.8244 (3)	0.0527 (11)	
H18A	0.4288	0.8499	0.8032	0.063*	
H18B	0.2471	0.8737	0.8146	0.063*	
C19	0.2579 (7)	0.7645 (3)	0.7669 (4)	0.0674 (14)	
H19A	0.2345	0.7779	0.7019	0.101*	
H19B	0.1568	0.7481	0.7870	0.101*	
H19C	0.3383	0.7243	0.7757	0.101*	

### Atomic displacement parameters (Å^2^)

**Table:**

	*U*^11^	*U*^22^	*U*^33^	*U*^12^	*U*^13^	*U*^23^
S1	0.0292 (5)	0.0334 (5)	0.0270 (5)	−0.0037 (4)	0.0063 (4)	−0.0028 (4)
S2	0.0311 (5)	0.0289 (5)	0.0283 (5)	−0.0037 (4)	−0.0004 (4)	0.0062 (4)
O1	0.0369 (15)	0.0395 (15)	0.0347 (14)	−0.0020 (11)	0.0097 (11)	−0.0078 (12)
O2	0.0370 (15)	0.0421 (16)	0.0548 (18)	−0.0075 (12)	0.0114 (13)	−0.0189 (14)
O3	0.0340 (15)	0.0321 (14)	0.0610 (18)	−0.0064 (11)	−0.0027 (13)	0.0199 (14)
O4	0.0427 (16)	0.0370 (15)	0.0471 (16)	−0.0134 (12)	0.0048 (13)	0.0007 (13)
O5	0.075 (2)	0.066 (2)	0.068 (2)	−0.011 (2)	0.0340 (19)	−0.0046 (19)
O1W	0.077 (3)	0.049 (2)	0.109 (3)	−0.0073 (19)	0.033 (2)	−0.017 (2)
N1	0.0402 (19)	0.0356 (18)	0.0403 (18)	0.0005 (14)	0.0066 (14)	0.0047 (15)
C1	0.0293 (19)	0.0285 (19)	0.0306 (19)	0.0029 (15)	0.0004 (15)	0.0008 (16)
C2	0.0290 (19)	0.0286 (18)	0.0312 (19)	0.0028 (14)	0.0034 (15)	0.0002 (16)
C3	0.0264 (18)	0.0294 (18)	0.0261 (17)	0.0026 (14)	0.0038 (14)	0.0017 (15)
C4	0.037 (2)	0.031 (2)	0.038 (2)	−0.0029 (16)	0.0060 (16)	−0.0062 (17)
C5	0.042 (2)	0.043 (2)	0.048 (2)	0.0028 (18)	0.0229 (19)	−0.006 (2)
C6	0.038 (2)	0.043 (2)	0.056 (3)	−0.0059 (18)	0.0198 (19)	−0.008 (2)
C7	0.032 (2)	0.035 (2)	0.050 (2)	−0.0036 (16)	0.0111 (17)	−0.0084 (19)
C8	0.0268 (18)	0.0259 (18)	0.0354 (19)	−0.0021 (14)	0.0116 (15)	−0.0018 (16)
C9	0.0260 (17)	0.0273 (18)	0.0276 (17)	0.0015 (14)	0.0095 (14)	0.0026 (15)
C10	0.0225 (17)	0.0258 (17)	0.0238 (16)	0.0048 (13)	0.0061 (13)	−0.0007 (14)
C11	0.0290 (18)	0.0268 (18)	0.0311 (19)	−0.0013 (14)	0.0034 (14)	0.0023 (16)
C12	0.038 (2)	0.0316 (19)	0.0288 (18)	0.0047 (16)	0.0087 (15)	0.0064 (16)
C13	0.039 (2)	0.041 (2)	0.0242 (18)	0.0041 (17)	0.0021 (15)	0.0026 (17)
C14	0.036 (2)	0.034 (2)	0.0294 (19)	−0.0019 (16)	0.0056 (15)	−0.0054 (17)
C15	0.048 (3)	0.065 (3)	0.037 (2)	−0.002 (2)	0.0095 (19)	0.002 (2)
C16	0.058 (3)	0.047 (3)	0.039 (2)	0.001 (2)	0.013 (2)	0.012 (2)
C17	0.047 (2)	0.033 (2)	0.050 (2)	−0.0032 (17)	0.0146 (19)	−0.0003 (19)
C18	0.063 (3)	0.047 (3)	0.047 (2)	−0.002 (2)	0.004 (2)	−0.004 (2)
C19	0.070 (3)	0.072 (3)	0.062 (3)	−0.019 (3)	0.014 (3)	−0.016 (3)

### Geometric parameters (Å, °)

**Table:**

S1—C3	1.791 (3)	C7—H7	0.9300
S1—S2	2.0528 (13)	C8—C9	1.498 (5)
S2—C10	1.793 (3)	C9—C10	1.399 (5)
O1—C1	1.212 (4)	C9—C14	1.400 (5)
O2—C1	1.316 (4)	C10—C11	1.398 (5)
O2—H2O	0.8400	C11—C12	1.387 (5)
O3—C8	1.266 (4)	C11—H11	0.9300
O4—C8	1.247 (4)	C12—C13	1.384 (5)
O5—C15	1.400 (6)	C12—H12	0.9300
O5—H5O	0.8401	C13—C14	1.381 (5)
O1W—H1W	0.8401	C13—H13	0.9300
O1W—H2W	0.8401	C14—H14	0.9300
N1—C16	1.473 (5)	C15—C16	1.499 (6)
N1—C17	1.479 (5)	C15—H15A	0.9700
N1—H1N	0.9000	C15—H15B	0.9700
N1—H2N	0.9000	C16—H16A	0.9700
C1—C2	1.485 (5)	C16—H16B	0.9700
C2—C3	1.394 (5)	C17—C18	1.491 (6)
C2—C7	1.395 (5)	C17—H17A	0.9700
C3—C4	1.399 (5)	C17—H17B	0.9700
C4—C5	1.381 (5)	C18—C19	1.526 (6)
C4—H4	0.9300	C18—H18A	0.9700
C5—C6	1.383 (6)	C18—H18B	0.9700
C5—H5	0.9300	C19—H19A	0.9600
C6—C7	1.369 (5)	C19—H19B	0.9600
C6—H6	0.9300	C19—H19C	0.9600
			
C3—S1—S2	105.07 (12)	C12—C11—H11	119.8
C10—S2—S1	105.81 (12)	C10—C11—H11	119.8
C1—O2—H2O	114.7	C13—C12—C11	120.8 (3)
C15—O5—H5O	105.7	C13—C12—H12	119.6
H1W—O1W—H2W	111.6	C11—C12—H12	119.6
C16—N1—C17	114.5 (3)	C14—C13—C12	118.9 (3)
C16—N1—H1N	108.6	C14—C13—H13	120.6
C17—N1—H1N	108.6	C12—C13—H13	120.6
C16—N1—H2N	108.6	C13—C14—C9	121.7 (3)
C17—N1—H2N	108.6	C13—C14—H14	119.2
H1N—N1—H2N	107.6	C9—C14—H14	119.2
O1—C1—O2	122.7 (3)	O5—C15—C16	113.4 (4)
O1—C1—C2	122.5 (3)	O5—C15—H15A	108.9
O2—C1—C2	114.7 (3)	C16—C15—H15A	108.9
C3—C2—C7	119.7 (3)	O5—C15—H15B	108.9
C3—C2—C1	121.1 (3)	C16—C15—H15B	108.9
C7—C2—C1	119.2 (3)	H15A—C15—H15B	107.7
C2—C3—C4	118.4 (3)	N1—C16—C15	111.7 (3)
C2—C3—S1	120.6 (3)	N1—C16—H16A	109.3
C4—C3—S1	121.1 (3)	C15—C16—H16A	109.3
C5—C4—C3	121.1 (3)	N1—C16—H16B	109.3
C5—C4—H4	119.4	C15—C16—H16B	109.3
C3—C4—H4	119.4	H16A—C16—H16B	108.0
C4—C5—C6	120.0 (4)	N1—C17—C18	113.5 (3)
C4—C5—H5	120.0	N1—C17—H17A	108.9
C6—C5—H5	120.0	C18—C17—H17A	108.9
C7—C6—C5	119.6 (4)	N1—C17—H17B	108.9
C7—C6—H6	120.2	C18—C17—H17B	108.9
C5—C6—H6	120.2	H17A—C17—H17B	107.7
C6—C7—C2	121.2 (4)	C17—C18—C19	111.6 (4)
C6—C7—H7	119.4	C17—C18—H18A	109.3
C2—C7—H7	119.4	C19—C18—H18A	109.3
O4—C8—O3	124.1 (3)	C17—C18—H18B	109.3
O4—C8—C9	120.2 (3)	C19—C18—H18B	109.3
O3—C8—C9	115.7 (3)	H18A—C18—H18B	108.0
C10—C9—C14	119.0 (3)	C18—C19—H19A	109.5
C10—C9—C8	122.6 (3)	C18—C19—H19B	109.5
C14—C9—C8	118.4 (3)	H19A—C19—H19B	109.5
C11—C10—C9	119.3 (3)	C18—C19—H19C	109.5
C11—C10—S2	120.7 (3)	H19A—C19—H19C	109.5
C9—C10—S2	120.0 (3)	H19B—C19—H19C	109.5
C12—C11—C10	120.4 (3)		
			
C3—S1—S2—C10	−87.44 (16)	O4—C8—C9—C14	−20.8 (5)
O1—C1—C2—C3	−2.9 (5)	O3—C8—C9—C14	158.1 (3)
O2—C1—C2—C3	178.7 (3)	C14—C9—C10—C11	0.4 (5)
O1—C1—C2—C7	178.1 (3)	C8—C9—C10—C11	179.5 (3)
O2—C1—C2—C7	−0.2 (5)	C14—C9—C10—S2	179.6 (3)
C7—C2—C3—C4	−1.0 (5)	C8—C9—C10—S2	−1.3 (4)
C1—C2—C3—C4	−180.0 (3)	S1—S2—C10—C11	15.5 (3)
C7—C2—C3—S1	179.9 (3)	S1—S2—C10—C9	−163.7 (2)
C1—C2—C3—S1	0.9 (5)	C9—C10—C11—C12	0.1 (5)
S2—S1—C3—C2	−166.8 (3)	S2—C10—C11—C12	−179.1 (3)
S2—S1—C3—C4	14.1 (3)	C10—C11—C12—C13	−0.8 (5)
C2—C3—C4—C5	0.9 (6)	C11—C12—C13—C14	0.9 (5)
S1—C3—C4—C5	−180.0 (3)	C12—C13—C14—C9	−0.4 (5)
C3—C4—C5—C6	−0.4 (6)	C10—C9—C14—C13	−0.2 (5)
C4—C5—C6—C7	−0.1 (7)	C8—C9—C14—C13	−179.3 (3)
C5—C6—C7—C2	0.0 (6)	C17—N1—C16—C15	175.9 (3)
C3—C2—C7—C6	0.5 (6)	O5—C15—C16—N1	53.8 (5)
C1—C2—C7—C6	179.5 (4)	C16—N1—C17—C18	175.8 (4)
O4—C8—C9—C10	160.1 (3)	N1—C17—C18—C19	−179.8 (4)
O3—C8—C9—C10	−21.0 (5)		

### Hydrogen-bond geometry (Å, °)

**Table:**

*D*—H···*A*	*D*—H	H···*A*	*D*···*A*	*D*—H···*A*
O2—H2o···O3^i^	0.84	1.70	2.535 (4)	178
N1—H1n···O4^ii^	0.90	2.10	2.887 (4)	146
N1—H2n···O1w^iii^	0.90	1.92	2.773 (5)	158
O5—H5o···O1^iv^	0.84	1.94	2.751 (5)	162
O1w—H1w···O4^v^	0.84	1.99	2.823 (5)	174
O1w—H2w···O3	0.84	2.26	3.036 (5)	154

Symmetry codes: (i) −*x*+3/2, *y*+1/2, −*z*+3/2; (ii) *x*+1/2, −*y*+3/2, *z*+1/2; (iii) *x*−1/2, −*y*+3/2, *z*+1/2; (iv) −*x*+1, −*y*+2, −*z*+2; (v) −*x*+1, −*y*+1, −*z*+1.
